# Filtering for increased power for microarray data analysis

**DOI:** 10.1186/1471-2105-10-11

**Published:** 2009-01-08

**Authors:** Amber J Hackstadt, Ann M Hess

**Affiliations:** 1 Center for Bioinformatics and Department of Statistics, Colorado State University, Fort Collins, CO, 80523, USA

## Abstract

**Background:**

Due to the large number of hypothesis tests performed during the process of routine analysis of microarray data, a multiple testing adjustment is certainly warranted. However, when the number of tests is very large and the proportion of differentially expressed genes is relatively low, the use of a multiple testing adjustment can result in very low power to detect those genes which are truly differentially expressed. Filtering allows for a reduction in the number of tests and a corresponding increase in power. Common filtering methods include filtering by variance, average signal or MAS detection call (for Affymetrix arrays). We study the effects of filtering in combination with the Benjamini-Hochberg method for false discovery rate control and q-value for false discovery rate estimation.

**Results:**

Three case studies are used to compare three different filtering methods in combination with the two false discovery rate methods and three different preprocessing methods. For the case studies considered, filtering by detection call and variance (on the original scale) consistently led to an increase in the number of differentially expressed genes identified. On the other hand, filtering by variance on the log_2 _scale had a detrimental effect when paired with MAS5 or PLIER preprocessing methods, even when the testing was done on the log_2 _scale. A simulation study was done to further examine the effect of filtering by variance. We find that filtering by variance leads to higher power, often with a decrease in false discovery rate, when paired with either of the false discovery rate methods considered. This holds regardless of the proportion of genes which are differentially expressed or whether we assume dependence or independence among genes.

**Conclusion:**

The case studies show that both detection call and variance filtering are viable methods of filtering which can increase the number of differentially expressed genes identified. The simulation study demonstrates that when paired with a false discovery rate method, filtering by variance can increase power while still controlling the false discovery rate. Filtering out 50% of probe sets seems reasonable as long as the majority of genes are not expected to be differentially expressed.

## Background

Microarrays allow researchers to examine the expression of thousands of genes simultaneously. The primary goal of many microarray experiments is to identify a group of genes that is differentially expressed between two or more conditions. Such "differentially expressed genes" (DEGs) are identified through statistical testing. With tens of thousands of genes represented on an array and one or more hypotheses being tested for each gene, a multiple testing adjustment is certainly warranted. For expression studies involving microarrays, it has become common practice to focus on control of the false discovery rate (FDR). The false discovery rate is the expected proportion of incorrect rejections among the rejected hypotheses. Let *V *be the number of truly null hypotheses that are rejected and *R *be the total number of hypotheses that are rejected. Let *Q *be defined as *V*/*R *when *R *> 0 and let *Q *= 0 if *R *= 0. FDR is then defined as FDR = E(*Q*) [1].

Many procedures are available for estimating or controlling FDR. Benjamini and Hochberg proposed an intuitive procedure for controlling FDR [1]. Storey and Tibshirani offer the q-value method to estimate the FDR [2]. The q-value is a measure of significance in terms of FDR. The q-value of a particular feature (gene) is the expected proportion of false positives among all features as extreme or more extreme than the observed one. The q-value method uses an estimate of *π*_0_, the proportion of p-values that correspond to tests in which the null hypothesis is true. Both the Benjamini-Hochberg and q-value methods are based on the assumption that the distribution of p-values corresponding to truly null hypotheses (the null distribution) follows a uniform distribution between zero and one. Additional FDR methods have been proposed by many authors, but we find the Benjamini-Hochberg and q-values methods to be the most commonly used methods.

FDR methods offer a substantial increase in power over methods that control family-wise error rate. However, low power can still be a problem when the proportion of differentially expressed genes is relatively low. In addition, researchers using standard manufactured arrays (i.e. Affymetrix GeneChips) have no control over the number of genes represented on the array. For example, the ATH1 (Arabidopsis) GeneChip contains approximately 22,500 probe sets, the MGU430 (mouse) GeneChip contains approximately 45,000 probe sets and the Wheat GeneChip contains roughly 61,000 probe sets. Hence, situations can arise where the number of tests is very large but the proportion of differentially expressed genes is relatively low, resulting in low power even when using an FDR method.

Filtering methods can be used to reduce the number of tests and therefore increase the power to detect true differences. An ideal filtering method would remove tests which are truly null (corresponding to genes that are equally expressed), while leaving those tests corresponding to genes which are truly differentially expressed. Several methods for filtering have been suggested including filtering by variance, signal, and MAS detection call.

All filtering methods discussed here can be applied without using information about treatment assignments. When filtering by variance, we remove genes with low variance across arrays (ignoring treatment). The rationale is that expression for equally expressed genes (EEGs) should not differ greatly between treatment groups, hence leading to small overall variance. The goal of filtering by signal is to filter out genes that have signal close to background level. Genes with low average signal (ignoring treatment) are removed. Filtering by MAS detection (or Present/Absent) call is a common choice of investigators using Affymetrix GeneChips. The MAS detection call algorithm is based on the use of the Wilcoxon Signed Rank test to compare PM (Perfect Match) and MM (Mismatch) probes within a probe pair. A "call" of Present, Absent or Marginal is made for each probe set [3]. The idea of filtering by detection call is that if a transcript is not present in any sample, then clearly it cannot be differentially expressed. Hence, we filter out probe sets that are called Absent on all arrays.

## Results

In order to evaluate the effect of filtering, we use three case studies as well as a simulation study. All programming was done in R using Bioconductor [4,5].

For the three case studies, we examine the effect of three filtering methods (variance, signal and detection call) as well as the results when no filtering is done. In order to facilitate direct comparisons between the filtering methods, we selected the same number of probe sets to be filtered out for all filtering methods.

Specifically, we found the number of probe sets not called Present on any array in a given experiment and hence filtered out by the detection call method. We then fix this to be the number of probe sets filtered out by the variance and signal filtering methods as well. In addition to the various filtering and FDR methods, we consider the RMA, MAS5 and PLIER methods for preprocessing. We note that all testing was done using expression values on the log_2 _scale. However, we examined the effect of filtering by variance on both the log_2 _and "original" scales. A 0.05 significance level was used for all methods.

For the simulation study, we start with simulated expression data and focus on the effect of filtering by variance. A 0.05 significance level was used for all methods.

### Case Study: Wheat Data

A study was conducted to examine gene expression of resistant and susceptible lines of wheat grown in the presence and absence of the Russian wheat aphid. The Affymetrix GeneChip Wheat genome array (containing 61,290 probe sets representing 55,052 transcripts for all 42 wheat chromosomes) was used for this study. RNA samples were collected from wheat plants in 2 × 2 factorial design. The design was originally balanced, but one array was dropped due to concerns about array quality. Each array represents a pooled sample from five seedlings. The data used here consists of 11 arrays: 3 arrays representing the resistant wheat variety in the absence of the Russian wheat aphid, 2 arrays representing the resistant wheat variety in the presence of the Russian wheat aphid, 3 arrays representing susceptible wheat variety in the absence of the Russian wheat aphid, and 3 arrays representing the susceptible wheat variety in the presence of the Russian wheat aphid.

For the purposes of this paper, we focus on two comparisons of interest: (1) comparison of gene expression of the resistant wheat line in the presence and absence of the Russian wheat aphid and (2) comparison of gene expression of the resistant and susceptible wheat lines in the absence of the Russian wheat aphid. These two comparisons were selected because the first is expected to yield a large number of DEGs while the second should yield fewer DEGs. Testing for the two comparisons of interest was performed using an analysis of variance (ANOVA) model and contrasts of factor level means.

In order to facilitate direct comparisons between the filtering methods, we selected the same number of probe sets to be filtered out for all filtering methods. A total of 30,234 probe sets (49%) were not called Present on any of the 11 arrays and were therefore filtered out by the detection call filtering method.

**Figure 1:**
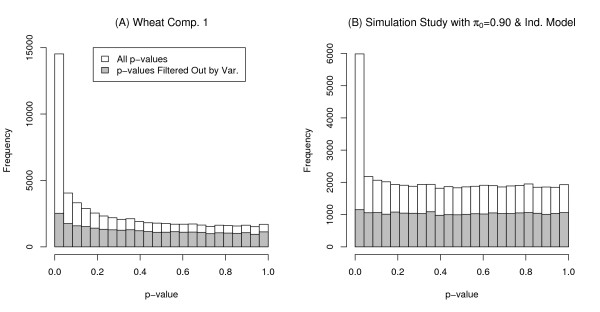
**Histogram of p-values for the Wheat case study and a simulation run**. Plot (A) is a histogram of p-values from wheat comparison 1 (using RMA preprocessing). The histogram of the p-values corresponding to the filtered low variance probe sets (lowest 49% variance on the log_2 _scale) are overlaid in gray. Plot (B) is the histogram of p-values from one run of the simulation (independent case with *π*_0 _= 0.90). A histogram of the p-values corresponding to the genes filtered out by variance (lowest 50% variance) are overlaid in gray.

Hence, when filtering by average signal (or variance), the probe sets with the smallest 30,234 average signal values (or variances) were filtered out. Figure 1A gives a histogram of p-values obtained from testing for DEGs for the first comparison with p-values corresponding to the filtered (low variance) probe sets overlaid in gray.

The number of probe sets corresponding to differentially expressed genes identified for each of the combinations of preprocessing (RMA, MAS5 and PLIER), filtering (none, MAS detection call, signal and variance on the log_2 _and original scales) and FDR methods (none, Benjamini-Hochberg, q-value) are shown in Table 1 for both wheat comparisons. We see that for a given preprocessing and FDR method, filtering by detection call, signal or variance (on the original scale) leads to an increase in the number of DEGs identified. In contrast, in some cases, filtering by variance on the log_2 _scale leads to a decrease in the number of DEGs identified (as compared to unfiltered data) for MAS5 and PLIER preprocessing methods.

**Table 1:** Number of DEGs Identified for Case Studies

			Filtering Method				
Case Study	Preprocessing Method	FDR Method	None	Detection Call	Variance (log_2_)	Variance (original)	Signal

Wheat Comp 1	RMA	None	15511	12524	12601	12932	12459
		BH	8546	9355	9464	9725	9163
		q-value	10333	12237	12369	13125	12233

Wheat Comp 1	MAS5	None	12547	10520	3497	10728	10539
		BH	5869	6896	1096	7088	6880
		q-value	6687	8968	1137	9305	8989

Wheat Comp 1	PLIER	None	17224	13127	8151	13992	13305
		BH	9713	9811	5436	10714	9913
		q-value	12167	13366	6010	15541	14242

Wheat Comp 2	RMA	None	10309	7454	7751	7776	7391
		BH	643	1593	1733	1704	1329
		q-value	1416	3390	3874	3791	3175

Wheat Comp 2	MAS5	None	8149	6187	2620	6353	6201
		BH	162	569	42	585	511
		q-value	328	1337	53	1485	1330

Wheat Comp 2	PLIER	None	10928	7473	5690	8150	7539
		BH	1048	1654	1219	2109	1633
		q-value	2067	3386	1736	4402	3366

Diabetes	RMA	None	3469	3043	2710	3018	2991
		BH	644	781	710	807	783
		q-value	728	892	778	904	884

Diabetes	MAS5	None	3097	2599	1555	2597	2595
		BH	412	481	239	484	478
		q-value	449	520	241	523	517

Diabetes	PLIER	None	3266	2895	1792	2912	2921
		BH	541	646	388	682	665
		q-value	615	765	440	793	759

Smoking	RMA	None	3414	2511	2155	2196	2146
		BH	126	155	143	135	107
		q-value	135	184	177	158	121

Smoking	MAS5	None	3684	2522	1843	2510	2506
		BH	112	145	109	146	137
		q-value	130	166	116	164	155

Smoking	PLIER	None	3097	1832	1824	1704	1643
		BH	64	80	78	77	70
		q-value	70	91	85	81	72

Table of number of probe sets identified as differentially expressed for each of the case studies. For each case study, we considered three preprocessing methods (RMA, MAS5 and PLIER), two FDR methods (Benjamini-Hochberg and q-value) and three filtering methods (MAS detection call, variance (on both the log_2 _and original scales) and signal). The stated significance level was 0.05 for all methods.

### Case Study: Diabetes Data

A study was conducted to examine gene expression in the cardiac left ventricle using a rodent model of diabetic cardiomyopathy [6]. The Affymetrix Rat GeneChip 230 2.0 array (with 31,099 probe sets) was used for this investigation. RNA samples were collected from the cardiac left ventricles of 7 diabetes induced rats and 7 controls. Each sample was hybridized to a single array. The data can be obtained from the NCBI Gene Expression Omnibus (accession number GSE5606) [7]. A two-sample t-test assuming equal variances was used to identify differentially expressed genes.

Similar to the analysis for the wheat data, we selected the same number of probe sets to be filtered out for all filtering methods. A total of 10,473 probe sets (34%) were called Absent on all 14 arrays and were therefore filtered out by the MAS detection call filtering method. Hence, the same number of probe sets were removed for the other filtering methods. The number of probe sets corresponding to differentially expressed genes for each of the combinations of preprocessing, filtering and FDR methods are found in Table 1. We see that for a given preprocessing and FDR method, filtering by detection call, signal or variance (on the original scale) leads to an increase in the number of DEGs identified. In contrast, filtering by variance on the log_2 _scale leads to a decrease in the number of DEGs identified (as compared to unfiltered data) for MAS5 and PLIER preprocessing methods.

### Case Study: Smoking Data

A study was conducted to examine gene expression in the lungs of young mice exposed to 14 days of cigarette smoke [8]. The Affymetrix Mouse Genome 430 2.0 array (with 45,101 probe sets) was used for this investigation. RNA samples were collected from the lungs of 6 mice exposed to cigarette smoke and 4 controls. Each sample was hybridized to a single array. The data can be obtained from the NCBI Gene Expression Omnibus (accession number GSE7310) [7]. A two-sample t-test assuming equal variances was used to identify differentially expressed genes.

A total of 19,471 probe sets (43%) were called Absent on all 10 arrays and were therefore filtered out by the MAS detection filtering method. Hence, the same number of probe sets were removed for the other filtering methods. The number of probe sets corresponding to differentially expressed genes for each of the combinations of preprocessing, filtering and FDR methods are found in Table 1. We see that for a given preprocessing and FDR method, filtering by detection call or variance (on the original scale) leads to an increase in the number of DEGs identified. In contrast, filtering by variance on the log_2 _scale leads to a decrease in the number of DEGs identified for MAS5 and PLIER preprocessing methods. We also observe a decrease in the number of DEGs identified when signal filtering is paired with RMA preprocessing.

### Simulation Study

We simulated expression data under two models: when signal values between genes are independent and when the signal values between genes follow a "clumpy dependence" [9]. The data was simulated to correspond to two groups of five samples (arrays) with signal values generated for 50,000 genes for each sample. We considered true *π*_0 _values of 0.7, 0.8, 0.9, 0.95, and 0.98. A total of 1000 runs were used for each simulation scenario.

The signal value for gene *g *in sample *k *in block *j *and group *i*, was generated according to the model

*Y*_*ijkg *_= *F*_*ig *_× *I*_*g *_+ *B*_*jk *_+ *Z*_*ijkg*_.

A proportion, *π*_0_, of genes were randomly selected to have indicator variable *I*_*g *_= 0 (corresponding to EEGs) and the rest of genes have *I*_*g *_= 1 (corresponding to DEGs). The term *F_*ig *_*~ *N *(1, 0.25^2^) for samples from one group only, thus giving the magnitude of the differential expression. To create the dependent simulation scenario ("clumpy dependence" among genes), genes were randomly grouped into 200 blocks of 250 genes, indicated by the subscript *j *and with *B*_*jk *_~ *N *(0, $\sigma_{b}^{2}$). The variable *Z*_*ijkg *_~ *N *(0, $\sigma_{g}^{2}$) where $\sigma_{g}^{2}$ ~ Uniform(u_min_, u_max_) was used to allow the variance to differ among genes. For the dependent case, $\sigma_{b}^{2}$ = 0.09, and for the distribution of $\sigma_{g}^{2}$, u_min _= 0.0, and u_max _= 0.18. For the independent case, $\sigma_{b}^{2}$ = 0, u_min _= 0.09, u_max _= 0.27. The values for $\sigma_{b}^{2}$, u_min_, and u_max _were chosen such that the distribution of the variance of *Y*_*ijkg *_is the same for both the dependent and independent models. Moreover, the distributions of *F*_*ig*_, *B*_*jk*_, and *Z*_*ijkg *_were selected so the distribution of p-values for the simulation study resembles the distribution of p-values seen in case studies. This is supported by the histogram of p-values shown in Figure 1.

For each run of the simulation, t-tests comparing the two groups were performed and the BH and q-value methods were applied, with and without filtering to the 50,000 resulting p-values. The t-tests were performed assuming equal variances for the two groups. Filtering was performed by variance, with the 25,000 genes with the lowest variances (ignoring group) being filtered out. An *α *= 0.05 level of significance was used for all FDR methods. A histograms of the p-values for a single run of the simulation with *π*_0 _= 0.9 for the independent case is shown in Figure 1B.

#### Power

**Figure 2:**
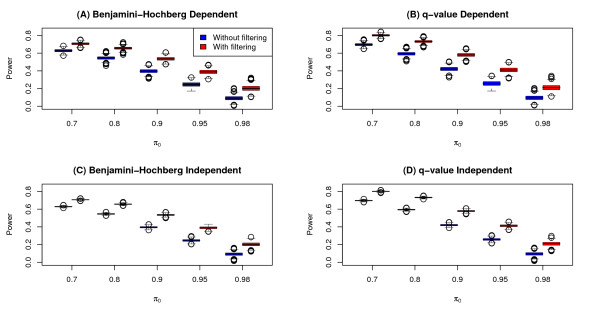
**Power results from simulation study**. Boxplots of observed power of tests for differential expression (0.05 significance level) for simulation runs both with and without variance filtering. Plots (A) and (B) are boxplots for simulation scenarios with a "clumpy dependence" among genes using the Benjamini-Hochberg (BH) and q-value methods, respectively. Plots (C) and (D) are boxplots for simulation scenarios modeling independence among genes using the BH and q-value methods, respectively.

The observed power for each method and each run was calculated as the proportion of true positives that were detected at the stated significance level of *α *= 0.05. The distribution of observed power for each of the FDR methods with and without filtering are shown in Figure 2 and summarized in Additional file 1 Table S1. As expected, the power for the two FDR methods increases as *π*_0 _decreases, demonstrating increased power as a higher proportion of genes are differentially expressed. More importantly, these results show that filtering by variance results in an overall gain in power for both FDR methods considered for both independent and dependent models. The gain in power due to filtering is fairly consistent across the range of *π*_0 _values. Not surprisingly, the power under the independent model was less variable than the corresponding power under the dependent model. However, the median power for a given value of *π*_0 _is about the same for independent and dependent models. Not unexpectedly (since BH is an FDR controlling procedure and therefore more conservative) we find that q-value has higher power than the Benjamini-Hochberg method for a given simulation scenario.

#### False Discovery Rate

**Figure 3:**
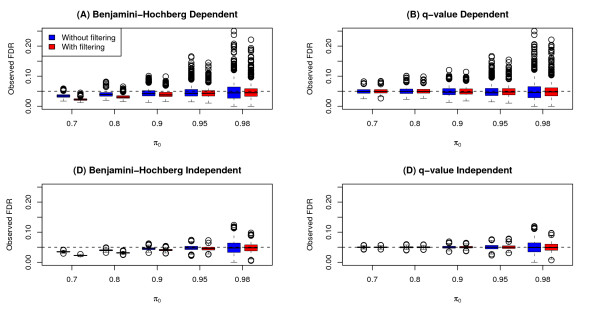
**FDR results from simulation study**. Boxplots of observed FDR of tests for differential expression for simulations runs both with and without variance filtering. Plots (A) and (B) are boxplots for simulation scenarios with a "clumpy dependence" among genes using the Benjamini-Hochberg (BH) and q-value methods, respectively. Plots (C) and (D) are boxplots for simulation scenarios modeling independence among genes using the BH and q-value methods, respectively. The nominal FDR level = 0.05 is represented by the horizontal dashed lines on plots.

The observed FDR for each method and each run was calculated as the proportion of false positives among the rejected hypotheses. This observed FDR was compared to the nominal FDR level of 0.05. The distribution of the observed false discovery rate for each of the simulation scenarios are shown in Figure 3 and summarized in Additional file 2 Table S2. The effect of filtering on the observed FDR is different for each of the FDR methods. For BH, the use of filtering actually leads to an overall decrease in observed FDR for lower values of *π*_0_. For q-value, the use of filtering has little effect on the observed FDR, except for some decrease in the variability of the simulation runs. All methods (with and without filtering) have median observed FDR less than or equal to the nominal level of *α *= 0.05. Similar to the results for power, the observed FDR of the simulation runs are more dispersed for the dependent model than for the independent model.

#### Analysis of Different Filtering Thresholds

**Figure 4:**
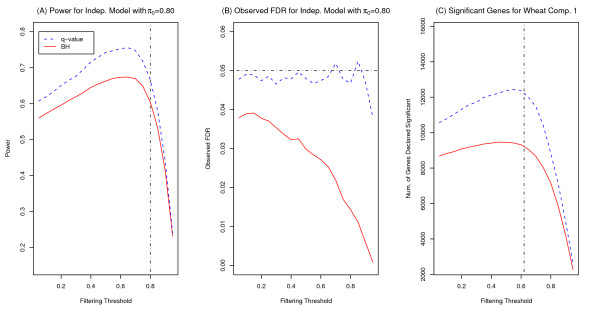
**Filtering by variance using different thresholds for wheat case study and simulation study**. Plot (A) is the observed power of one run of simulation (independent case with *π*_0 _= 0.8 represented by the vertical dashed line) found when filtering by variance using different variance quantiles (0.05 to 0.95) as thresholds and controlling FDR at *α *= 0.05. Plot (B) is the observed FDR of the same simulation run found when filtering by variance using different variance quantiles. The horizontal dashed line represents the nominal FDR. Plot (C) is the number of differentially expressed genes (DEGs) identified for Wheat comparison 1 (using RMA preprocessing) and filtering by variance (on the log_2 _scale) using different variance quantiles (0.05 to 0.95) as thresholds. The vertical dashed line represents the q-value estimate of the proportion of null hypotheses ($\hat{\pi }$_0 _= 0.62).

We examined the effect of different thresholds when filtering by variance. The observed power and FDR for a simulation run of the independent model with *π*_0 _= 0.80 across a range of variance quantiles (ranging from 0.05 to 0.95) is shown in Figure 4A and 4B. For instance, if the variance quantile is 0.10, then 10% of genes (with the lowest variances) are filtered out for BH and q-value methods.

For both FDR methods, the power increases as an increasing proportion is filtered out (corresponding to an increasing quantile) until the proportion (quantile) gets close to *π*_0_. At the same time, the observed FDR for these methods stays close to or below the *α *level of 0.05. As the quantile used for the threshold becomes close to *π*_0_, the power begins to decrease. This suggests that we are starting to remove genes that are truly differentially expressed. Hence both the BH and q-value methods have improved power (while still maintaining a desirable FDR level) if filtering is done at a level somewhat close to, but well below, *π*_0_. Similar results were obtained for the dependent models.

We also examined the effect of filtering with different thresholds for the three case studies. The number of DEGs found when varying the proportion of genes filtered out for wheat comparison 1 (using RMA preprocessing paired with filtering by variance on the log_2 _scale) is also shown in Figure 4C. For this comparison, the number of DEGs identified gradually increased for both Benjamini-Hochberg and q-value methods as the proportion filtered out increased until a threshold of about 0.60. The quantile at which the number of DEGs began to decrease is close to the q-value estimate of *π*_0 _($\hat{\pi }$_0 _= 0.62). Similar results were seen for the other case studies and preprocessing methods, but these results are not shown here.

## Discussion

McClintick and Edenberg previously studied the effects of filtering by MAS detection call and signal in combination with MAS5 and RMA preprocessing methods [10]. They recommend filtering out probe sets that are not called Present in at least 50% of samples in at least one treatment group. When using signal as a filtering criteria, they filtered out probe sets that did not have average signal greater than some threshold in at least one treatment group. Instead of filtering out probe sets that are not called Present in at least 50% of samples for at least one treatment group, we filtered out probe sets that were not called Present for any samples. A benefit of this method is that no knowledge of treatment assignments is used for filtering. In addition, in our experience, for moderately sized experiments (20 arrays or less) this method removes the vast majority of probe sets that would be removed using the 50% rule. However, as the number of arrays increases, it becomes more likely that a probe set corresponding to a truly unexpressed transcript will be called Present on at least one array just by chance. Hence we could see more dramatic differences between the two methods for larger experiments.

In their analysis, McClintick and Edenberg found filtering by MAS detection call to be superior to filtering by signal because it results in decreased FDR. Their logic for filtering out Absent called genes is clear, "Data for genes not actually expressed represent experimental noise and cannot increase true positives, but can (and do) generate false positives." While this is true, we must bear in mind that the MAS detection call is itself a statistical test and the truth of which genes are unexpressed is unknown. In addition, filtering by MAS detection call is not an option for spotted cDNA arrays or other types of manufactured arrays besides Affymetrix GeneChips.

We consider three different filtering methods in combination with two FDR methods and three preprocessing methods. For all case studies, preprocessing methods and FDR methods examined, filtering by detection call and variance (on the "original scale") increased the number of DEGs identified when compared to unfiltered data. In one case, filtering by signal (when paired with RMA preprocessing) lead to a decrease in the number of DEGs identified. In most cases, filtering by variance on the log_2 _scale in combination with MAS5 and PLIER methods actually lead to a decrease in the number of DEGs identified. This is surprising since testing was conducted on the log_2 _for all methods.

We believe that there are two factors contributing to this counterintuitive result. First of all, there is a relationship between average signal and variance and, for MAS5 and PLIER, the direction of this relationship depends on the scale. Based on the case studies considered, the correlation between average signal and variance for MAS5 ranged between -0.48 and -0.72 on the log_2 _scale and between 0.69 and 0.74 on the original scale. For PLIER, the correlation ranged between -0.31 and -0.74 on the log_2 _scale and between 0.15 and 0.47 on the original scale. For RMA, the correlation ranged between 0.14 and 0.28 on the log_2 _scale and between 0.33 and 0.61 on the original scale. One reason the log_2 _transformation is used is to stabilize the variance. However, it seems that for MAS5 and PLIER, this transformation over corrects and leads to increased variance for low expression transcripts. The result is that on log_2 _scale, high expression genes tend to have relatively low variances.

In addition to the relationship between signal and variance, there is a tendency for high expression genes to be over-represented in the list of DEGs. To examine this, we calculated the proportion of DEGs (using a significance level of 0.05 without filtering or applying any multiple testing adjustment) that had average signal in the top 50%. Hence, if there was no relationship between average signal and significance, we would expect 50% of DEGs to have average signal in the top 50%. The actual proportions varied by case study and preprocessing method ranging between 45% and 84%. In only one case (PLIER applied to the smoking data), was this percentage less than 50%.

These relationships between signal and variance and signal and significance lead to removal of high expression genes when using the MAS5 or PLIER methods and filtering by variance on the log_2 _scale. Since highly expressed genes are more likely to be identified as DEGs, then this filtering method tends to filter out genes that are likely to be differentially expressed. Filtering by variance on the original scale works better for these methods, even when testing is done on the log_2 _scale. This can be seen by examining the histogram of p-values corresponding to those genes filtered out by variance (not shown). The distribution of p-values more closely approximates a uniform distribution when filtering by variance is done on the original scale for MAS5 and PLIER. We suggest that whatever filtering method researchers choose, they examine the distribution of p-values corresponding to those genes filtered out.

Filtering by detection call and variance (on the original scale) consistently led to an increase in the number of differentially expressed genes identified. This was true for both cases where a large proportion of genes are differentially expressed (i.e. wheat comparison 1) and a small proportion of genes are differentially expressed (i.e. Smoking data). However, we note that for other data sets we examined we were not able to identify any DEGs (using a multiple testing adjustment) either with or without filtering. It is possible that some of these are cases where no genes are differentially expressed. On the other hand, it could be that even after filtering, the power was still too low. Either way, if no DEGs were identified to begin with, there is certainly no harm in attempting filtering.

The simulation study focuses on filtering by variance. We note that the simulated data does not exactly mimic observed microarray results. Specifically, we did not consider the relationships between signal and variance and signal and significance. In addition, the simulation study applies filtering by variance on the same scale as testing and does not represent a specific preprocessing method. Because of these issues, there may be concerns about the generalizability of the simulation results. The key issues for extending the simulation results are the full distribution of p-values, the null distribution of p-values and the distribution of filtered out p-values. Regarding the full distribution of p-values, we choose simulation parameters to generate realistic distributions. Regarding the null distribution of p-values, we examined simulation scenarios that represented both dependent and independent cases. Regarding the distribution of filtered out p-values, we note that for both the case studies and the simulation, there were significant departures from the uniform distribution based on the Kolmogorov-Smirnov test (data not shown). Specifically, for all case studies, preprocessing methods and filtering methods, the K-S test rejected the assumption of uniformity (of the filtered out p-values) at the 0.05 significance level. For the simulations studies, the assumption of uniformity (of the filtered out p-values) was rejected more than 5% of the time at the 0.05 significance level (i.e. for *π*_0 _= 0.9 case, the assumption was rejected for 45% of independent runs and 82% of dependent runs). However, the departures from the uniform distribution seemed to be larger for the observed data.

Based on our simulation study, we find that filtering by variance results in increased power without an increase in the observed FDR when paired with BH or q-value methods. While only filtering by variance was used in the simulation study, it is expected that similar results could be found if filtering by detection call had been explored. This is supported by the large overlap in the number of probe sets identified by both the variance and detection call filtering methods for the case studies. Based on the three case studies examined, the percentage overlap in DEGs identified using detection call and variance filtering was consistently above 80% for all preprocessing methods and FDR methods (data not shown). This is based on variance filtering on the original scale for MAS5 and PLIER, but on both the original and log_2 _scales for RMA.

While filtering by MAS detection call leads to some natural thresholds (i.e., filtering out probe sets which are not called Present on any array), it is not clear how to choose a threshold when filtering by variance. For the simulation, we removed 50% of the genes. As long as the majority of genes are not differentially expressed, then this seems like a reasonable choice. When we examined the effect of varying the proportion filtered out, we found that the power increased until the proportion filtered out approached *π*_0_. A similar effect was observed for the case studies when using $\hat{\pi }$_0 _from the q-value method. Since a common assumption of microarray analysis is that the majority of genes will not be differentially expressed, filtering 50% of the values should be reasonable in most cases. As an example, when we filter out 50% of values by variance for the Diabetes data (for which *π*_0 _is estimated to be between 0.77 and 0.88 depending on preprocessing method) we see consistent gains in the number of DEGs identified as compared to the values presented in Table 1 (data not shown).

The filtering methods examined in this paper can be applied to data with any number of treatment groups. We note that in cases when there are three or more treatment groups, the global F-test could also be used for filtering. Specifically, those genes which do not pass the F-test would be removed from further testing (i.e. pairwise comparisons). A concern with this method is the need to control the overall error rate. Since false rejections when performing the F-test will affect false rejections when performing further testing, the FDR of the whole procedure must be controlled. Jiang and Doerge suggest a two-step procedure to control the overall FDR [11]. Though the two-step procedure is only appropriate for experiments involving three or more treatment groups, if there are more than three treatment groups, it becomes very complex because the possible configurations of means of the factor levels must be determined to apply the two-step procedure.

In this paper, we focus on the use of filtering to increase the number of differentially expressed genes identified in gene expression studies when using an FDR method. However, not all researchers use FDR to identify a group of differentially expressed genes. Recently, the MicroArray Quality Control (MAQC) project concluded "that a straightforward approach of fold-change ranking plus non stringent P cutoff can be successful in identifying reproducible gene lists" [12]. We believe that this method of identifying DEGs by using a p-value cutoff followed by ranking genes by absolute fold change can be improved by considering the false discovery rate. In particular, an estimate of the FDR can aid in the selection of an appropriate significance cutoff, one that will help control the number of false positives.

## Conclusion

The need for the multiple testing adjustments to microarray data is well established. However, after applying an FDR method, the number of differentially expressed genes that are identified in the analysis is often greatly reduced and when the number of true DEGs is small relative to the number of tests, applying a multiple testing adjustment can result in a substantial loss in power. In this paper we examine the effect of filtering out probe sets in order to increase power. Three filtering criteria were considered: MAS detection call, variance, and average signal. Our analysis also considered the performance of two FDR methods (Benjamini-Hochberg and q-value) and three preprocessing methods (RMA, MAS5 and PLIER).

For the case studies considered, filtering by detection call and variance (on the original scale) consistently led to an increase in the number of DEGs identified. On the other hand, filtering by variance on the log_2 _scale had a detrimental effect when paired with MAS5 and PLIER preprocessing methods, even when the testing was done on the log_2 _scale. For a fixed preprocessing and FDR method, the DEGs identified with filtering by detection call and variance filtering (on the original scale for MAS and PLIER or either scale for RMA) were largely the same.

While we saw an increase in the number of DEGs identified for the case studies when filtering by variance was used in combination with an FDR method, we cannot determine whether this is due to an increase in power or false discovery rate. Hence a simulation study was performed to examine the issues of power and false discovery rate. The simulation study demonstrates that filtering by variance (with the median of the variances of the genes as a threshold) improves the power over a range of null proportions for the two FDR methods considered. The q-value method has higher power than BH in all the cases considered both with or without filtering. The observed FDR is maintained close to or below the stated level for both FDR procedures. Overall, filtering by variance can effectively increase power while maintaining the stated FDR and performs especially well when paired with q-value method.

Finally, we examined the effect of various thresholds for variance filtering. We found that filtering out 50% of probe sets seems reasonable as long as the majority of genes are expected to be equally expressed. This assumption can be checked based on the estimate of *π*_0 _provided by the q-value method.

## Methods

### Preprocessing Methods

All preprocessing was carried out in R using BioConductor. MAS5 [3] and RMA [13] expression indices were calculated using the *affy *package [14]. PLIER [15] expression indices were calculated using the *plier *package. We note that RMA and PLIER expression indices are calculated on the log_2 _scale, so when we discuss the "original" scale for those methods, values have been transformed using *f*(*x*) = 2^*x*^.

### FDR Methods

Benjamini and Hochberg proposed a simple adjustment to the p-values from hypotheses tests to control the overall false discovery rate. Suppose one is testing m hypotheses resulting in m p-values. Let *p*_(1) _≤ *p*_(2) _≤ ⋯ ≤ *p*_(*m*) _be the ordered p-values with the corresponding hypotheses *H*_(1) _≤ *H*_(2) _≤ ⋯ ≤ *H*_(*m*)_. Let *k *be the largest *i *such that $p_{\left(i\right)}\leq \frac{i}{m}\alpha$. By rejecting the hypotheses, *H*_(*i*)_, for *i *= 1,..., *k*, the FDR is controlled at level *α *[1]. Benjamini-Hochberg adjusted p-values were calculated using the *multtest *package [16].

For a specific feature (gene), the q-value is the expected proportion of false positives among all features as extreme or more extreme than the one observed. Suppose one is testing m hypotheses and obtains m p-values, *p*_1_, *p*_2_,..., *p*_*m*_, corresponding to these m hypotheses. If we assume that p-values are uniformly distributed under the null hypothesis, then an estimate of the FDR is given by:

(1) $$F\hat{D}R(t)=\frac{{\hat{\pi }}_{0}\cdot m\cdot t}{\#\{p_{i}\leq t\}}.$$

where *t *is the level (threshold) at which you would like to control FDR, $\hat{\pi }$_0 _is an estimate for the proportion of truly null hypothesis. The q-value of a feature *i *is estimated as

(2) $${\hat{q}}_{i}(p_{i})=\underset{t\geq p_{i}}{\min}F\hat{D}R(t).$$

The *qvalue *package [17] was used to calculate q-values.

We note that BH is an FDR controlling procedure (providing an upperbound on FDR), while q-value is an FDR estimation method. Because of this, BH is more conservative than the q-value method in most situations. This is reflected in Figures 2 and 3, where for a given simulation scenario, both the power and observed FDR tend to be lower for BH as compared to q-value. A more thorough comparison and discussion of these two FDR methods (as well as others) can be found in [9].

### Filtering Methods

Three methods for filtering were considered in our analysis. If a probe set was "filtered out" by a particular method, the p-value for that probe set was not passed through to the FDR method and it could not be called differentially expressed.

When filtering by variance, the variance of signal values (ignoring treatment assignments) is calculated for each probe set. Probe sets are then ranked by variance, and the probe sets falling below some threshold are filtered out. For the simulation study, 50% of probe sets were filtered (except where otherwise noted). We note that for the case studies, filtering by variance was done on both the original and log_2 _scales.

When filtering by signal, the mean signal (ignoring treatment assignments) is calculated for each probe set. Probe sets are then ranked by mean signal, and the probe sets falling below some threshold are filtered out. We note that average signal was calculated on the log_2 _scale.

Filtering using the MAS detection call only applies when using Affymetrix arrays. For each probe set on each array, a detection call of Present, Absent or Marginal is made. The detection call is based on the Wilcoxon signed rank test performed using PM and MM values. Detection calls were made using the *affy *package [14]. For both the case studies and the simulation study, probe sets that were never called Present on any array (sample) were filtered out.

## Abbreviations

BH: Benjamini-Hochberg; EEG: equally expressed gene; DEG:differentially expressed gene; FDR: false discovery rate

## Authors' contributions

AMH and AJH designed the study and helped to draft the manuscript. AJH did all of the programming.

## Supplementary Material

Additional file 1 **Detailed Statistics on Power from Simulation Study.** The table displays the 5th, 25th, 50th, 75th, and 95th quantiles of the observed power for simulation runs. Power was calculated with and without variance filtering for both FDR methods(Benjamini-Hochberg and q-value) using a stated FDR level of 0.05. Both dependent and independent cases were considered over a range of *π*_0 _values, where *π*_0 _is the proportion of genes which are truly not differentially expressed. Click here for file

Additional file 2 **Detailed Statistics on FDR from Simulation Study**. The table displays the 5th, 25th, 50th, 75th, and 95th quantiles of observed FDR for simulation runs. Observed FDR was calculated with and without variance filtering for both FDR methods (Benjamini-Hochberg and q-value) using a stated FDR level of 0.05. Both dependent and independent cases were considered over a range of *π*_0 _values, where *π*_0 _is the proportion of genes which are truly not differentially expressed. Click here for file
